# Characterization of milk and soy phospholipid liposomes and inflammation in 3T3-L1 adipocytes

**DOI:** 10.3168/jdsc.2020-0054

**Published:** 2021-06-19

**Authors:** Erica Kosmerl, Israel García-Cano, Diana Rocha-Mendoza, Rafael Jiménez-Flores

**Affiliations:** Department of Food Science and Technology, The Ohio State University, Columbus 43210

## Abstract

Milk phospholipids (PL) are valuable dairy components that appear to impart human health benefits, including improved cognitive function in infants and adults. The commercial food industry uses primarily plant-based sources of PL, such as soy lecithin. However, it remains unclear whether different compositions of PL from different dietary sources, such as milk, convey the same benefits. We hypothesized that PL derived from bovine milk or soy have differing physiological effects in terms of inflammation due to their differences in composition. The objectives of this study were to characterize milk and soy liposomes by their physicochemical properties and composition and to evaluate their effects in vitro by means of inflammatory gene expression analyses. Milk and soy phospholipid large unilamellar vesicles (MPL-LUV and SPL-LUV, respectively) prepared using thin-film hydration coupled with extrusion were similar in terms of structure, size, and stability; however, they differed significantly in composition. The 3T3-L1 adipocytes were selected for this work because adipocytes are the main site of uptake, synthesis, modification, and breakdown of lipids and are important inflammatory mediators in mammalian systems. In this work, these cells exposed to both liposome varieties showed high biocompatibility and low cytotoxicity up to concentrations of 0.5 mg/mL as measured by colorimetric MTT and lactate dehydrogenase assays. Furthermore, SPL-LUV showed trends toward stimulating inflammation compared with MPL-LUV as measured by expression of 2 proinflammatory cytokines, monocyte chemoattractant protein-1 (MCP-1) and IL-6. Expression of MCP-1 significantly increased 1.82-fold relative to the control upon SPL-LUV treatment, with similar trends for IL-6 (increased 1.59-fold). The MPL-LUV showed relatively no change in cytokine expression. The results obtained in this work suggest that the methodology used to prepare LUV and the composition and proportion of milk PL are important in measuring cell physiology changes and inflammatory status in mammalian cells.

## Highlights

• Biological activities of phospholipids in mammalian cells were measured using an objective method.
• We present a standardized, reproducible procedure to prepare liposomes from phospholipids.
• This method shows how to measure and evaluate liposomes from different sources of phospholipids.
• This work assesses the viability and toxicity of different sources of phospholipids and demonstrates a measurable physiological reaction to lipid stimuli.

## Graphical Abstract

**Figure:**
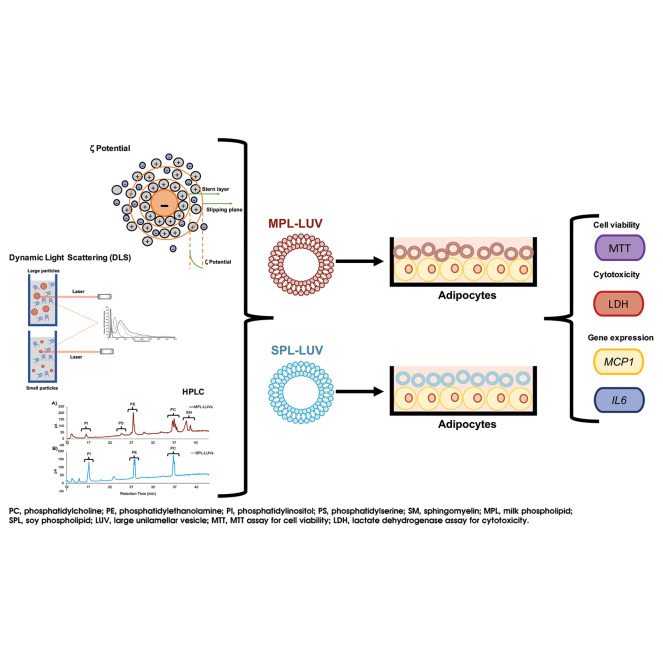
**Summary:** Because of the differing composition between milk and soy phospholipids, they may influence human health differently. We present a method to assess the biological activities of these unique sources of phospholipids using mammalian cell culture. In doing so, we standardized a procedure to generate liposomes with similar physicochemical properties (i.e., particle size and zeta potential), despite their inherent differences in composition. Our data suggest that milk and soy phospholipid liposomes may have differing effects on mammalian cell physiology and potentially human health.

Phospholipids (**PL**) serve multifunctional roles within biological systems and mammalian cells. They (1) serve as a substrate for energy storage; (2) facilitate compartmentalization between 2 distinct aqueous environments; (3) act as signaling messengers and bioactive molecules; and (4) form interactions with other membrane components, such as glycoproteins (Nicolson and Ash, 2014). Dysfunction of plasma membrane lipids is associated with aging, oxidation, and several chronic diseases, such as cancer and Alzheimer's disease (Nicolson et al., 2016; Korshavn et al., 2017). As supported by several clinical trials, dietary PL can restore membrane damage via lipid replacement therapy and aid in membrane protection (Nicolson and Ash, 2014). However, it remains unclear whether the source, and therefore composition, of PL is important for these perceived benefits on health.

One natural source of dietary PL is the milk fat globule membrane (**MFGM**), which emulsifies and stabilizes the fat globules in milk. The MFGM is biosimilar in PL composition to the mammalian cell membrane as it is originally derived from the secretory cell membrane in breast tissue (Keenan et al., 1970; Ortega-Anaya and Jiménez-Flores, 2019). The MFGM-derived milk PL (**MPL**), notably sphingomyelin (**SM**), have gained significant attention in recent years for their bioactive activities and numerous benefits to human health. These include their roles in neurodevelopment, gut health, cholesterol absorption, lipid metabolism, and inflammation, deepening the link between dietary lipids and human health (Rombaut and Dewettinck, 2006; Tanaka et al., 2013; Norris and Blesso, 2017). The predominant MPL and their relative proportions in bovine milk include phosphatidylcholine (32%; **PC**), phosphatidylethanolamine (23%; **PE**), phosphatidylserine (7%; **PS**), phosphatidylinositol (9%; **PI**), and SM (29%; Kosmerl et al., 2021). In contrast to MPL, soy PL (**SPL**) or soy lecithin are more readily incorporated into food products due to their low cost and widespread availability. However, the PL composition of SPL differs greatly from that of MPL. The predominant PL in soy include PC (10–15%), PE (9–12%), PI (8–10%), and PS (1–2%; Küllenberg et al., 2012). Unlike MPL, SPL are very low in PS and absent of SM, which can only be obtained from animal sources, such as bovine milk. These differences in composition suggest that MPL and SPL may dissimilarly influence health.

In general, PL are relatively insoluble in aqueous solutions; however, structures known as liposomes can be used to mimic the structural complexity of PL in nature and improve PL solubility in aqueous solutions. Liposomes are self-assembled, spherical structures composed of at least 1 PL bilayer that encapsulates an aqueous core. Of the various classifications of liposomes, large unilamellar vesicles (**LUV**) are liposomes ranging from 100 nm to 1 mm that can be produced to generate vesicles of uniform size (Emami et al., 2016).

Given the innate differences in PL composition between milk and soy sources, we aimed to assess and compare the physicochemical properties of liposomes derived from MPL and SPL and examine their influence on the expression of proinflammatory cytokines. The LUV of both MPL (**MPL-LUV**) and SPL (**SPL-LUV**) were prepared via thin-film hydration. One hundred milligrams of MPL (NZMP Phospholipid Concentrate 700, Fonterra Co-Operative Group) or SPL (Ultralec-P, ADM) was dissolved in 4 mL of 2:1 chloroform:methanol solution in a round-bottom flask. Organic solvents were evaporated off using a rotary evaporator (Rotavapor R-210, Buchi Corporation) at 45°C for 30 min to produce an evenly distributed thin film. The thin films were hydrated with agitation using sterile 1× Dulbecco's PBS (Corning Life Sciences) at 55°C for 2 to 3 h. Particle size and lamellarity were controlled by extrusion with a mini extruder (200-nm pore size) at 55°C (T&T Scientific Corp.) with 11 passes. Final PL content of the liposomes was quantified using the Stewart assay (Stewart, 1980).

The LUV were characterized by dynamic light scattering and phase analysis light scattering zeta potential to assess similarity in terms of particle size, polydispersity index, and surface charge for both liposome dispersions. For all measurements, liposomes were diluted in PBS to meet the turbidity requirements of the instrument (NanoBrook ZetaPALS Potential Analyzer; Brookhaven Instruments Corporation) and were measured at a 90° angle at 37°C with an equilibration time of 100 s. Particle size measurements were performed in triplicate for 5 min using the refractive index, viscosity, and dielectric constant of water (1.329, 0.692 cP, and 73.91, respectively) as well as the reported refractive index of liposomes (1.45; Thompson et al., 2006). The zeta potentials of the same liposome dispersions were measured using a surface zeta potential electrode. Five measurements of 30 cycles each at 37°C were performed for each zeta potential measurement. Reported data represent the average of 3 individual LUV preparations.

High-performance liquid chromatography (Dionex Ultimate 3000 UHPLC System, Thermo Scientific) combined with a charged aerosol detector (Dionex Corona Veo RS, Thermo Scientific) were then used to quantify PL composition of liposomes after Bligh-Dyer extraction (Bligh and Dyer, 1959). The chromatographic conditions using a binary solvent system were adapted from Braun et al. (2010) with some modifications. Major PL class separation was achieved on 2 identical Syncronis silica columns (4.6 mm × 250 mm, 5-μm particle size) with a guard column of the same packing (4.6 mm × 10 mm, 5-μm particle size) from Thermo Scientific. Mobile phase A consisted of 3.0 g/L ammonium acetate, and mobile phase B consisted of 100 + 3 (vol/vol) acetonitrile–methanol. A 52-min gradient run separated the major classes of PL using the following linear gradient conditions: 5% A and 95% B at time (t) = 0 min; 5% A and 95% B at t = 2 min; 25% A and 75% B at t = 35 min; 25% A and 75% B at t = 40 min; 5% A and 95% B at t = 41 min; and 5% A and 95% B at t = 52 min. A flow rate of 1.0 mL/min was used, and the column oven was held at 55°C. Quantification via peak integration was calculated using calibration curves of PC, PE, PS, PI, and SM standards (PH-9-1KT, Millipore-Sigma).

The 3T3-L1 preadipocytes (ATCC CL-173TM; American Type Culture Collection) were maintained in preadipocyte media [high-glucose Dulbecco's modified Eagle medium (**DMEM**) and 10% bovine newborn calf serum] in a 37°C, 5% CO_2_ humidified incubator. Preadipocytes plated for experiments were grown to 100% confluency and grown for an additional 48 h before initiating differentiation with differentiation media [high-glucose DMEM, 10% heat-inactivated fetal bovine serum (**FBS**), 1.0 μg/mL bovine insulin, 0.5 m*M* methylisobutylxanthine, and 1.0 μ*M* dexamethasone]. After 48 h, the medium was continually replaced with adipocyte maintenance media (DMEM, 10% FBS, and 1.0 μg/mL insulin) and used for experiments on d 7 to 8 of differentiation. These cells were selected due to their role in metabolic syndrome and obesity. Future experiments depend on evidence of response of these cells to LUV.

Cell viability and cytotoxicity after liposome treatment was assessed using the colorimetric MTT assay (MTT Assay Kit, Millipore-Sigma) and lactate dehydrogenase (**LDH**) assay (Cytotoxicity Detection Kit LDH, Roche Diagnostics), respectively. Differentiated 3T3-L1 adipocytes were treated with 5% FBS medium mixed with a vehicle control (PBS) or 0.05, 0.25, 0.5, or 1.5 mg/mL MPL-LUV or SPL-LUV. Positive and negative controls included 1% Triton-X 100 or PBS, respectively. After 24 h of treatment, the cell supernatants were removed and transferred to a new 96-well plate for the LDH assay, and cells were washed twice with PBS. Then, 100 μL of 5% FBS in DMEM and 10 μL of 5 mg/mL MTT reagent were added to each well and incubated at 37°C for 2 h. The purple precipitate was solubilized through addition of 50 μL of dimethyl sulfoxide (Corning) to each well, and then 150 μL of PBS was added. The absorbance was measured using a Multiskan GO plate reader at 570 nm (test wavelength) and 630 nm (reference wavelength). A conservative cut-off value of 80% was selected for this assay based on the International Organization for Standardization recommendations (ISO, 2009). Three independent experiments were performed.

For the LDH assay, the cell supernatants were centrifuged at 117 × *g* for 10 min. The LDH reagent was prepared according to the manufacturer's instructions (Cytotoxicity Detection Kit LDH, Roche Diagnostics). Ten microliters of cell supernatant, 40 μL of PBS buffer, and 50 μL of LDH reagent were added to a new 96-well plate and incubated at 25°C for 30 min. Absorbance at 490 nm (test wavelength) and 620 nm (reference wavelength) was measured in a plate reader. A cytotoxicity cut-off of 5% was selected as a conservative value based on previous literature (Vinken and Blaauboer, 2017). Data were collected from 3 individual experiments.

To investigate the role of MPL-LUV and SPL-LUV on inflammation, serum-starved adipocytes were treated with 0.5 mg/mL LUV or PBS for 24 h. Cells were then washed twice and incubated in DMEM for 6 h, and TRI reagent (Millipore-Sigma) was used to isolate RNA following the manufacturer's protocol. Reverse transcription was performed using the iScript Reverse Transcription Kit (Bio-Rad), and quantitative PCR was subsequently performed with iQ SYBR Green Supermix on a Bio-Rad CFX96 Touch PCR system following the manufacturer's protocol. The genes analyzed included *RPLP0* (reference gene), monocyte chemoattractant protein-1 (*MCP1*), and *IL6* using primer sequences from Cranmer-Byng et al. (2015). Relative gene expression was normalized using CFX software (Bio-Rad) under the ΔΔCq method. Three individual experiments were performed. For all data, statistical analysis by either a Student's *t*-test or 1-way ANOVA with post hoc Tukey test when appropriate was performed using JMP Pro 14 software (SAS Institute Inc.).

The MPL-LUV and SPL-LUV obtained had similar effective diameters around 230 nm, slightly above the extruder membrane pore size of 200 nm (Table 1). This result may be explained by the phenomenon in which ellipsoid vesicles pass through the extruder membrane more easily than do spherical vesicles (Lesieur et al., 1991; Berger et al., 2001). The polydispersity indices were near 0.1, indicating uniform, monomodal dispersions for both MPL-LUV and SPL-LUV (Table 1). In addition, the zeta potential of MPL-LUV and SPL-LUV was −15.63 ± 3.19 and −27.71 ± 2.67 mV, respectively (Table 1). In general, zeta potential values farther from zero (irrespective of charge) are indicative of greater electrostatic repulsion and increased stability (Salopek et al., 1992). Although similar in stability, the slight difference in zeta potential between milk and soy sources is likely attributed to the differences in PL composition of the liposome dispersions, as composition can influence not only surface charge but also membrane permeability and resistance to environmental factors (Thompson and Singh, 2006). The MPL-LUV prepared in this study had surface charges similar to those of vesicles produced from MFGM-derived MPL via sonication (Liu et al., 2013) and milk fat globules prepared from fresh bovine milk (Singh, 2006).

**Table 1:** Physicochemical properties and phospholipid (PL) quantification of milk (MPL) and soy (SPL) large unilamellar vesicles (LUV)

Item	Physicochemical property			Polar lipid species (mg/100 mg of total PL)				
	Effective diameter (nm)	Polydispersity index	Zeta potential (mV)	PC	PE	PI	PS	SM
MPL-LUV	229.3 ± 7.0	0.127 ± 0.019	−15.63 ± 3.19	27.90 ± 1.81	26.46 ± 1.80	6.80 ± 0.33	8.60 ± 3.34	30.24 ± 0.60
SPL-LUV	235.3 ± 28.4	0.118 ± 0.031	−27.71 ± 2.67	36.15 ± 0.72	17.06 ± 0.51	46.79 ± 0.21	—	—

^1^PC = phosphatidylcholine; PE = phosphatidylethanolamine; PI = phosphatidylinositol; PS = phosphatidylserine; SM = sphingomyelin.

Table 1 also shows the PL composition of MPL-LUV and SPL-LUV, in which both types of vesicles contained PC, PE, and PI; SM and PS were present in MPL-LUV and undetected in SPL-LUV. In addition, the relative abundance of PI was much greater in the soy samples compared with milk. These values are in accordance with reported values for the composition of MPL and SPL. Specifically, the composition of the Fonterra MPL was previously reported to contain 33.68, 23.91, 10.12, and 32.29 g of PC, PE, PI, and SM, respectively, per 100 g of PL (Morifuji et al., 2017). Furthermore, the SPL-LUV composition is in accordance with soy lecithin values reported in the literature. Scholfield (1981) reported soy lecithin containing 19 to 21% PC, 8 to 20% PE, and 20 to 21% PI as a percent of total components. Although similar data were obtained, some variation can result from quantification methods, biological and seasonal variation, and means of extraction (Rombaut and Dewettinck, 2006). Taken together, these data suggest that MPL-LUV and SPL-LUV produced by thin-film hydration and 200-nm extrusion are physically similar in terms of structure, population distribution, and stability despite their known chemical differences in PL composition.

The visible effect of MPL-LUV and SPL-LUV on adipocytes is shown in Figure 1A. The cells treated with MPL-LUV and with PBS (control) appeared similar in terms of shape, size, and form; however, cells treated with SPL-LUV appeared irregular and nonuniform after 24 h of incubation. To assess the physiological concentration range of LUV in fully differentiated adipocyte cell culture, cell viability (MTT) and cytotoxicity (LDH) experiments were performed as, thus far, no standard concentration for assessment of liposomes or delivery of PL in vitro has been established. The MTT assay assessed LUV concentrations ranging from 0.05 to 1.5 mg of PL/mL of medium for both MPL-LUV and SPL-LUV (Figure 1B). Both liposome varieties had viability measures above 80% for concentrations of 0.05, 0.25, and 0.5 mg of PL/mL. Conversely, cell viability at a concentration of 1.5 mg of PL/mL for both milk and soy decreased below 75% to 72.5 and 55.9% cell viability, respectively, suggesting potential incompatibility at very high concentrations. The changes in MTT cell viability from MPL-LUV and SPL-LUV treatment were then compared with the LDH assay measurement of cytotoxicity. The cytotoxicity data from MPL-LUV and SPL-LUV treatment are shown in Figure 1C. The mean cytotoxicity values of the 0.05, 0.25, and 0.5 mg/mL concentrations of MPL-LUV or SPL-LUV were below 2%, suggesting no cytotoxic effects of LUV at these concentrations. The cytotoxicity levels increased with 1.5 mg/mL of MPL-LUV and SPL-LUV to 7.7 and 11.6% cytotoxicity, respectively. These data from both the MTT and LDH assays show similar trends of high biocompatibility of liposomes in adipocyte cell culture. Concentrations up to 0.5 mg/mL of either MPL-LUV or SPL-LUV are within the suitable concentration range of this cell culture model. Therefore, we selected a concentration of 0.5 mg/mL LUV for further analysis on the inflammatory response.

**Figure 1:**
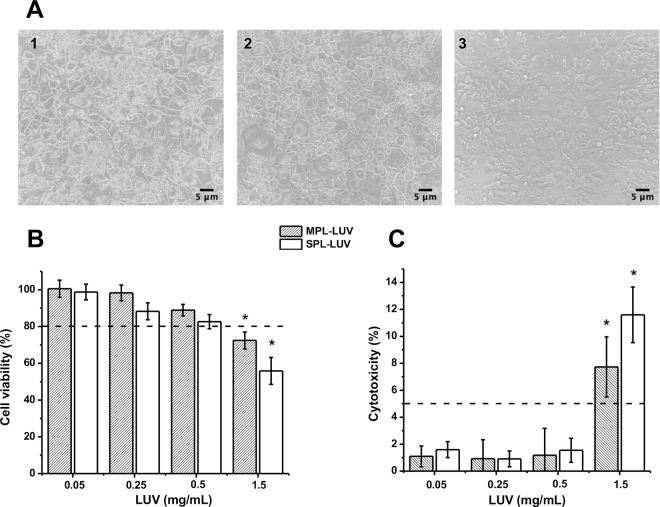
(A) Brightfield micrographs of adipocytes treated with (1) PBS, (2) 0.5 mg/mL milk phospholipid (MPL) large unilamellar vesicles (LUV), or (3) 0.5 mg/mL soy phospholipid (SPL) LUV for 24 h using 20× magnification. (B) Cell viability (MTT assay) and (C) cytotoxicity (lactate dehydrogenase assay) of 3T3-L1 adipocytes in response to various concentrations of milk or soy liposome treatment. Dotted lines represent cut-off values. Asterisk indicates significant difference between treatment and cut-off values (Student's *t*-test, *P* < 0.05). Error bars represent the mean ± SD of 3 independent experiments.

Additionally, we assessed the effect of milk and soy liposomes on the physiological response of adipocytes with respect to gene expression of 2 inflammatory cytokines: MCP-1 and IL-6. Figure 2A shows the increase in expression of MCP-1 by 1.82-fold in response to SPL-LUV treatment relative to the vehicle control; MPL-LUV did not significantly influence MCP-1 expression. MCP-1 is a well-studied chemokine responsible for the infiltration of monocytes and macrophages into a given infection site or tissue and is upregulated in obesity (Deshmane et al., 2009; Panee, 2012). Changes in IL-6 expression showed similar trends with respect to the MCP-1 data (Figure 2B). The SPL-LUV increased IL-6 expression relative to the vehicle control, albeit not at a statistically significant level, whereas MPL-LUV did not change IL-6 gene expression. Although IL-6 plays a multifunctional role in that it acts as both a proinflammatory and anti-inflammatory mediator, the combined increases in IL-6 and MCP-1 in response to SPL-LUV suggest the promotion of an inflammatory state (Scheller et al., 2011).

**Figure 2:**
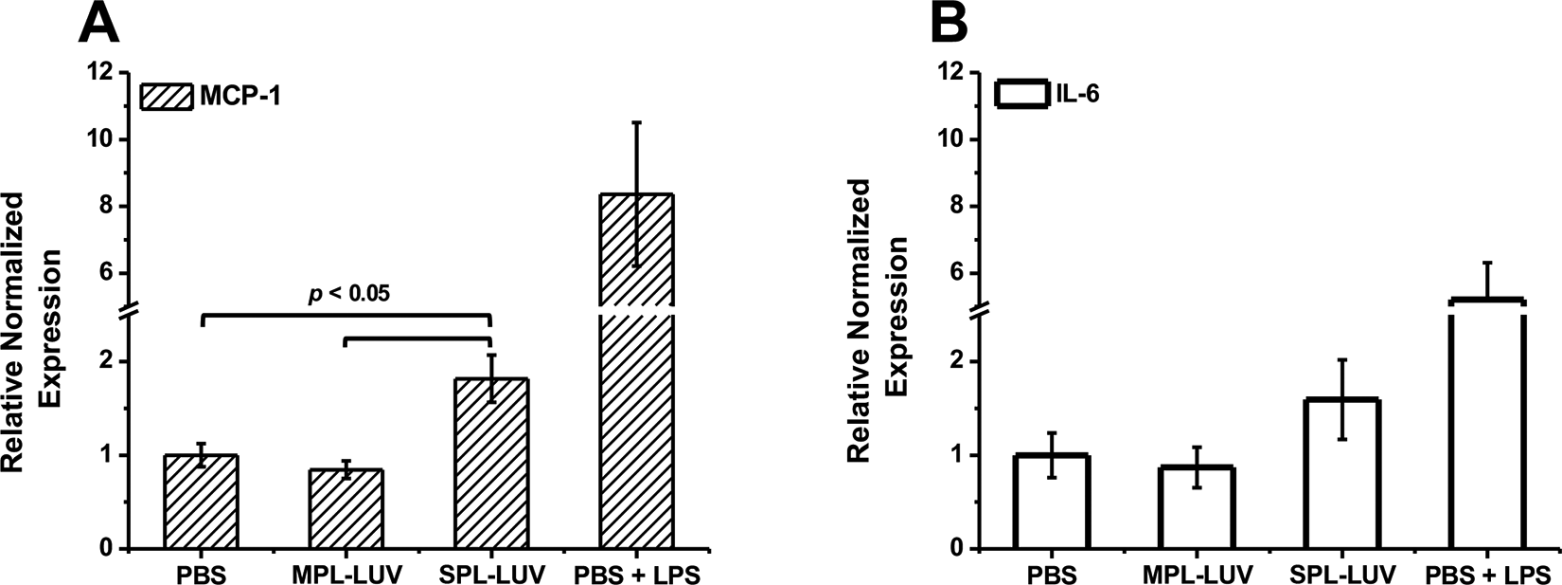
Relative gene expression of inflammatory cytokines (A) monocyte chemoattractant protein-1 (MCP-1) and (B) IL-6 in response to 24-h treatment with milk phospholipid large unilamellar vesicles (MPL-LUV) or soy phospholipid large unilamellar vesicles (SPL-LUV). PBS = vehicle control; PBS + LPS = positive control. Error bars indicate mean ± SEM (n = 3).

As shown previously, the key differences between MPL-LUV and SPL-LUV in this study were in composition and, in particular, SM content. Bovine SM has been reported to reduce systemic inflammation in mice fed high-fat diets and to attenuate UV-B-induced inflammation in keratinocytes (Oba et al., 2015; Norris et al., 2017). Other individual PL classes, such as PC, have also been shown be anti-inflammatory regulators in rheumatoid arthritis and colitis (Castro-Gómez et al., 2015). Taken together with the current study, these findings suggest that not only the inherent composition but also the ratio of PL classes may play an integral part in the immune-modulating properties of dietary PL. These results show a tendency for soybean PL to favor inflammation and a clear effect on the shape of mammalian cells. However, a broader investigation into the effects of MPL and SPL on inflammation using additional proinflammatory and anti-inflammatory cytokines along with additional cell models is required. Furthermore, this work opens opportunities for various challenge studies after priming cells with these PL.
